# Targeting DNA helicase CMG complex and NFκB2-driven drug-resistant transcriptional axis to effectively treat KRAS^G12D^-mutated pancreatic cancer

**DOI:** 10.1186/s40164-025-00669-w

**Published:** 2025-05-26

**Authors:** Jeffrey Xiao, Joshua Kim, Brandon Park, David J. Baylink, Cedric Kwon, Victoria Tran, Scott Lee, Kevin Codorniz, Laren Tan, Pamela Lobo Moreno, Amy Schill-Depew, Saied Mirshahidi, David De Semir, Diana Hanna, Kiran Naqvi, Huynh Cao, Chien-Shing Chen, Joanne Xiu, Heinz-Josef Lenz, Hamid Mirshahidi, Mark E Reeves, Yi Xu

**Affiliations:** 1 Division of Discovery, Innovation and Regenerative Medicine, Department of Medicine, School of Medicine, Loma Linda University, Loma Linda, 92354 CA USA; 2 Division of Endocrinology, Diabetes & Metabolism, Department of Medicine, School of Medicine, Loma Linda University, Loma Linda, 92354 CA USA; 3 Division of Pulmonary, Critical Care, Hyperbaric and Sleep Medicine, Department of Medicine, School of Medicine, Loma Linda University, Loma Linda, 92354 CA USA; 4 Biospecimen Laboratory, Department of Medicine and Basic Sciences, School of Medicine, Loma Linda University, Loma Linda, 92354 CA USA; 5 Division of Hematology and Oncology, Department of Medicine, School of Medicine, Loma Linda University, Loma Linda, 92354 CA USA; 6 Loma Linda University Cancer Center, Loma Linda, 92354 CA USA; 7 Caris Life Sciences, Inc, Phoenix, 85040 AZ USA; 8 Norris Comprehensive Cancer Center, Keck School of Medicine, University of Southern California, Los Angeles, 90033 CA USA; 9 Division of Hematology and Oncology, Department of Medicine, Chao Family Comprehensive Cancer Center, University of California Irvine, Irvine, CA 92868 USA

**Keywords:** PDAC, NFκB2, KRAS, MRTX1133, Bedaquiline, Helicase, Mitochondrion, ATP, DDIT, S100P

## Abstract

Pancreatic ductal adenocarcinoma (PDAC) is a devastating malignancy with a 5-year overall survival (OS) rate of approximately 12%. More than 90% of PDAC patients harbor oncogenic mutations in the Kirsten rat sarcoma viral homolog (*KRAS)* gene. MRTX1133 (MRTX), a novel inhibitor of *KRAS*^*G12D*^ (the most common *KRAS* mutation found in pancreatic and colon cancers) has shown promise as a therapeutic agent. To address reported resistance to MRTX, we adapted our anti-leukemia co-targeting strategy and evaluated a combination of MRTX and Bedaquiline (BED), an FDA-approved inhibitor of mitochondrial ATP production, in in vitro human PDAC models. The combination of MRTX and BED demonstrated enhanced cytotoxic effects by disrupting all 11 genes within the DNA helicase family (CMG complex: *CDC45-MCM-GINS*), which are essential for initiating DNA replication and regulating cell cycle progression. Notably, real-world data analysis from Caris Life Sciences and NCI-TCGA database revealed that low transcriptomic expression of the DNA helicase CMG complex was significantly associated with prolonged survival (e.g., low *CDC45* expression and low *GINS2* expression with greater than 8 months longer overall survival) in PDAC patients with *KRAS*^*G12*^ mutations (*N* = 9,717; *P* < 0.00001). However, this combination therapy also triggered strong pro-survival nuclear reprogramming. This effect was mediated by significant genetic activation of an NFκB2-DDIT (DNA damage-induced transcript) axis, which supported tumor chromosomal integrity and DNA repair mechanisms. To overcome NFκB2-driven resistance mechanisms, we explored a triple-targeting strategy that addresses metabolic and genomic plasticity in addition to actively intercepting cell division. This approach combines MRTX1133, Bedaquiline, and the NFκB2 inhibitor SN52, offering a novel therapeutic avenue to treat aggressive pancreatic cancer and potentially improve patient outcomes.

**Supplementary Information:**

The online version contains supplementary material available at 10.1186/s40164-025-00669-w.

To the editor

## Background

Pancreatic ductal adenocarcinoma (PDAC) is among the deadliest cancers, with limited treatment options available [1]. Over 90% of PDAC patients possess *KRAS* gene mutations, which are also present in approximately 30% lung cancers and 50% colon cancers [2]. Treatment resistance, unclear refractory mechanisms, and a lack of prognostic biomarkers worsen survival outcomes in PDAC patients [3]. To identify effective treatments, we adapted our previous anti-leukemia metabolism strategy [4] for PDAC, a malignancy requiring active metabolic reprogramming for local and systemic progression [5].

## Results

**Fig. 1:**
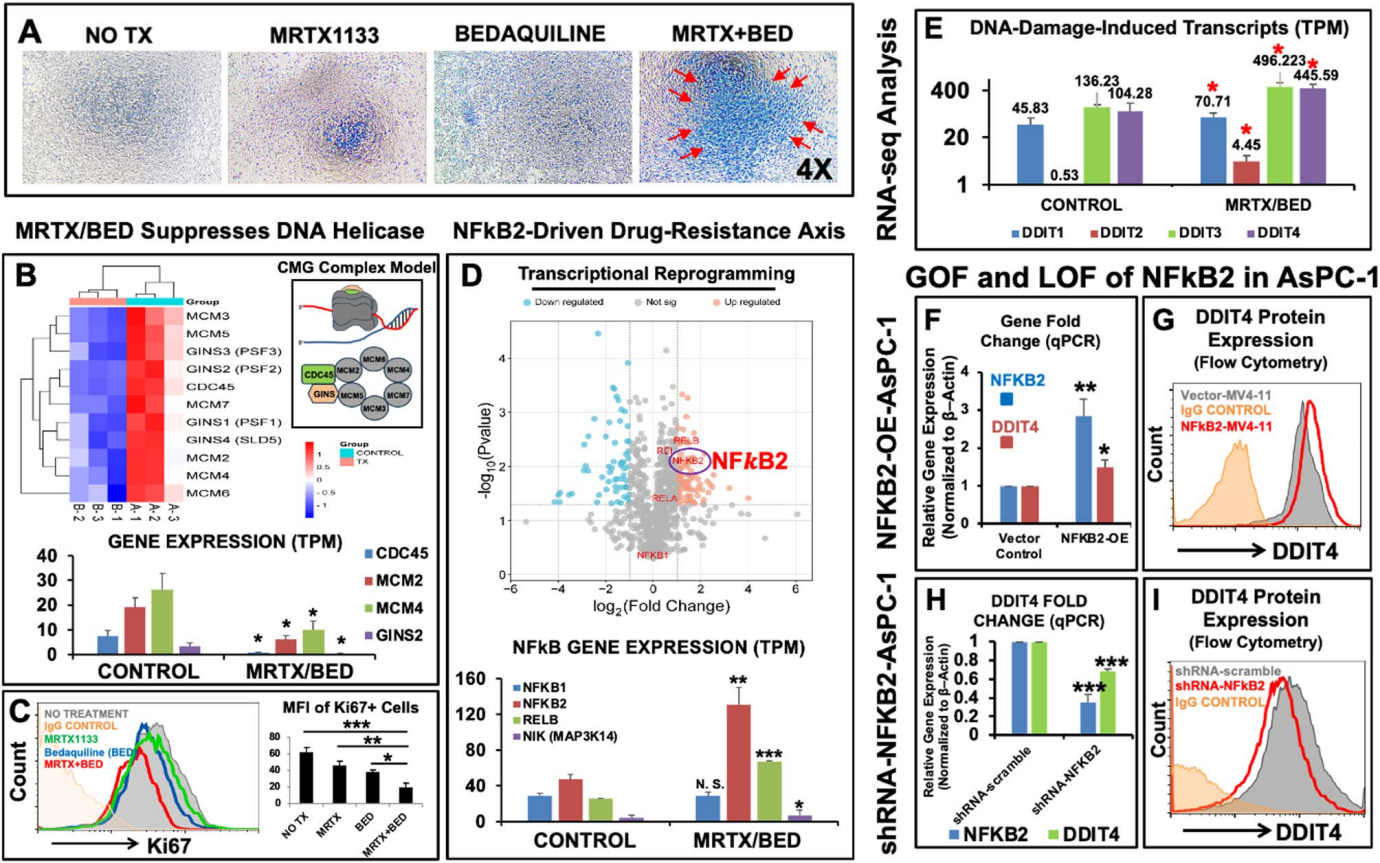
The combination of MRTX1133 (MRTX) and Bedaquiline (BED) effectively treats human pancreatic cancer cells with KRAS^G12D^ mutation (AsPC-1 cells) in vitro **A)** Representative phase-bright images of trypan blue-stained cells across different experimental groups (treated for 48 h). Blue-stained cells indicate dead or dying cells. Red arrows highlight the larger population of dead cells in the MRTX/BED treatment group compared to non-treatment (Control) groups, 100 nM MRTX-treated, and 20 µM BED-treated groups **B)** Heatmap of RNA-seq data comparing gene expression levels of CMG complex (DNA replicative helicase) family members between MRTX/BED combination (TX) groups and non-treatment (Control) groups. Redder color blocks represent higher expression levels, while bluer color blocks indicate lower expression levels. **Right insert**: Schematic structure (Drosophila model) of the CMG helicase unwinding duplex DNA. ***Lower panel***: Cumulative RNA-seq data (transcripts per million, TPM, the absolute abundance of transcripts) showing significantly decreased expression of *CDC45*,* MCM2*,* MCM4*, and *GINS2* genes in MRTX/BED combination (TX) groups versus non-treatment (Control) groups **C)** Representative flow cytometry (FC) histogram plot showing Ki67 expression across treatment groups, including 100 nM MRTX-treated (green line), 20 µM BED-treated (blue line), 100 nM MRTX + 20 µM BED (red line), non-treatment (NO-TX, filled grey line), and IgG-fluorescent control (orange line). **Right panel**: Cumulative data of mean fluorescence intensity (MFI) levels showing significantly decreased Ki67 expression in MRTX/BED combination (TX) groups compared to non-treatment (Control) groups, 100 nM MRTX-treated, and 20 µM BED-treated groups **D)** Volcano map (RNA-seq) showing differentially expressed genes (DEGs) between MRTX/BED combination (TX) groups and non-treatment (Control) groups. The X-axis represents log2-transformed fold changes, while the Y-axis represents log10-transformed significance values. Orange dots indicate upregulated DEGs; blue dots indicate downregulated DEGs; and grey dots indicated non-significant DEGs. The purple cycle highlights the upregulated expression of NFκB2. ***Lower panel***: Cumulative RNA-seq data (TPM) showing significantly increased expression of *NFκB2*,* RELB* and *NIK (MAP3K14)* genes in MRTX/BED combination (TX) groups compared to non-treatment (Control) groups. *NFκB1* gene expression showed no significant changes **E)** Cumulative RNA-seq data (TPM) showing significantly increased expression of various DDIT (DNA damage-induced transcripts) genes in MRTX/BED combination (TX) groups compared to non-treatment (Control) groups **F)** Gene expression analysis of *NFκB2* and *DDIT4 (Redd1)* in NFκB2-overexpressed (OE) AsPC-1 cells using qPCR. mRNA expression data show significantly increased expression (fold change normalized to *β-actin*) of *DDIT4 (Redd1)* in NFκB2-OE AsPC-1 compared to Vector-Control ASPC-1 cells **G)** Representative flow cytometry (FC) histogram plot showing increased DDIT4 (Redd1) expression in NFκB2-OE AsPC-1 cells (red line) compared to Vector-Control AsPC-1 cells (filled grey line), and IgG-fluorescent control (orange line) **H)** Gene expression analysis of *NFκB2* and *DDIT4 (Redd1)* following NFκB2 knockdown using shRNA in AsPC-1 cells. qPCR data show significantly decreased expression (fold change normalized to *β-actin*) of *DDIT4 (Redd1)* in shRNA-NFκB2 AsPC-1 compared to shRNA-scramble/control AsPC-1 cells **I)** Representative FC histogram plot showing decreased DDIT4 (Redd1) expression in NFκB2-shRNA knockdown AsPC-1 cells (red line) compared to Vector-Control AsPC-1 cells (filled grey line), and IgG-fluorescent control (orange line) Where applicable, data are presented as means ± SEM. Statistical significance: N.S. (not significant), *p < 0.05, **p < 0.01, ***p < 0.005, *N* = 3. Statistical analysis: two-tailed unpaired t test and one-way ANOVA

**Fig. 2:**
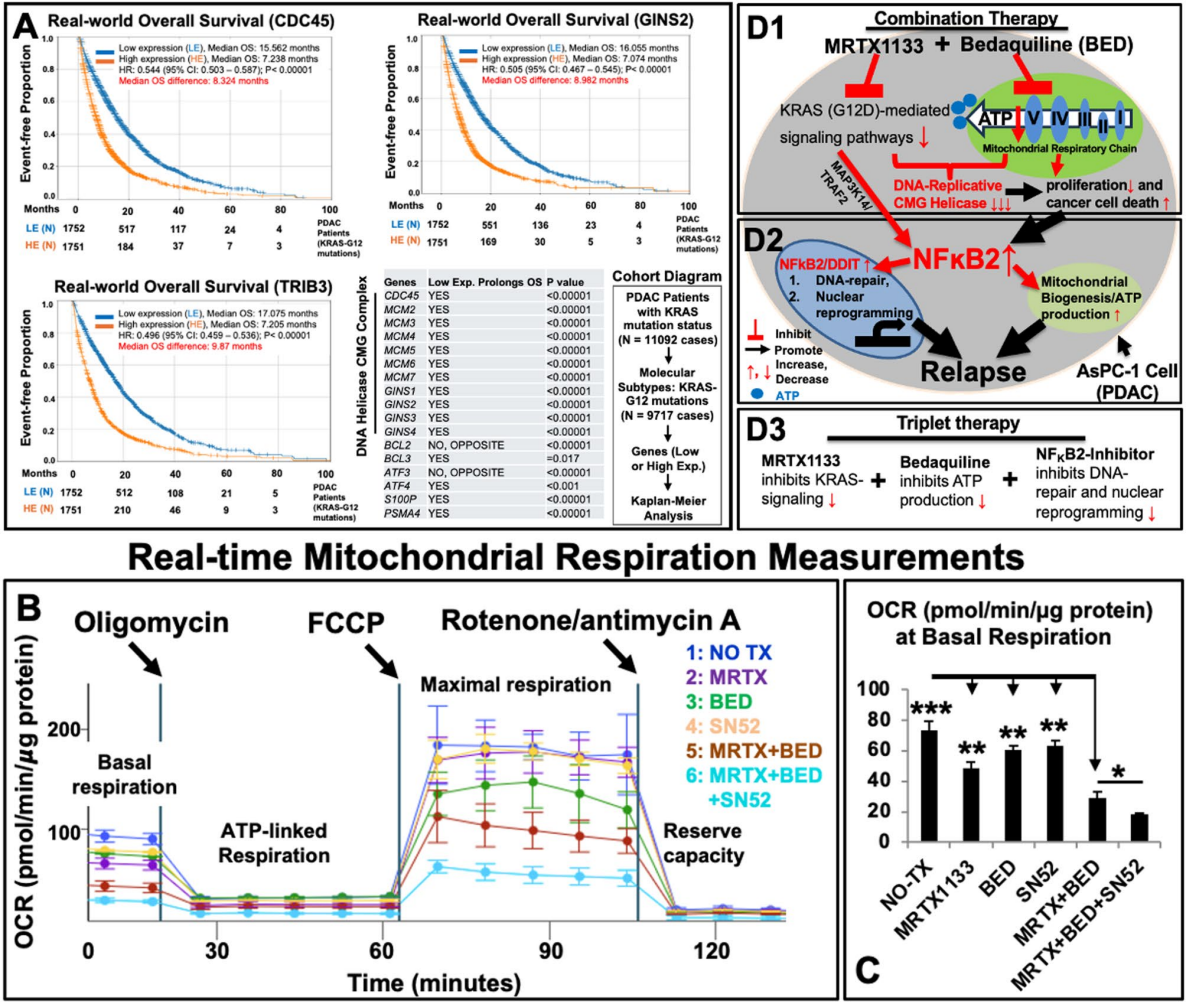
MRTX113/Bedaquiline-based therapies have the potential to improve PDAC patient survival outcomes by inhibiting the DNA helicase CMG complex and NFκB2-driven axis **A)Plots**: Real-world overall survival (OS) analysis of pancreatic cancer patients (*N* = 1,752) from Caris Life Sciences with low transcriptomic expression of *CDC45*,* GINS2*, and *TRIB3* (tribbles pseudokinase 3, an NFκB2-inducible gene) genes compared to patients (*N* = 1,751) with high expression levels of the same genes. Results show significantly improved event-free survival in patients with low gene expression levels **Table**: Checklist of prognostic genes whose low expression levels (Low Exp.) are potentially associated with prolonged OS in pancreatic cancer patients **Cohort Diagram**: From the total cohort of PDAC patients with *KRAS* mutation status (*N* = 11,092), those harboring *KRAS*^*G12*^ mutations (*N* = 9,717), including variants such as G12D, G12C, and G12V, were selected for further analyses of real-world OS Statistical analysis: Kaplan-Meier curves were generated for survival estimates. Hazard ratios (HR) were computed using the Cox proportional hazards model. Significant differences in survival times were assessed with the log-rank test **B)** Mitochondrial oxygen consumption rate (OCR) in AsPC-1 cells treated for 48 h under different experimental conditions was analyzed using the Seahorse XFe24 Analyzer (Agilent). Treatment groups included: (1) NO TX (non-treated control), (2) 100 nM MRTX1133 (MRTX), (3) 20 µM Bedaquiline (BED), (4) 15 µM SN52, (5) 100 nM MRTX + 20 µM BED, and (6) 100 nM MRTX + 20 µM BED + 15 µM SN52 **C)** Cumulative Seahorse data of basal OCR readings across different treatment groups of AsPC-1 Real-time traces and averaged data of OCR revealed that oxygen usage devoted to ATP production was significantly decreased in both combination and triplet therapies at the basal respiratory stage and subsequent drug-stimulated stages of Seahorse measurements, compared to single-agent regimens and non-treated controls **D)** Overview of targeting NFκB2-mediated DNA-repair and nuclear reprograming to overcome MRTX-resistant relapse in PDAC cells. **D1)** Mitochondria-targeted drugs such as Bedaquiline (BED), an ATP synthase blocker (Complex V), enhance the therapeutic efficacy of MRTX1133 (MRTX) in treating *KRAS*^*G12D*^-mutated PDAC cells by suppressing DNA replicative helicase activity. This leads to inhibited proliferation and increased cell death. However, treatment stress and mitochondrial damage activate the pro-survival transcription factor NFκB2 in AsPC-1 cells **D2)** Increased NFκB2 promotes the nuclear expression of genes such as *DDIT1 (GADD45α)* and *DDIT4 (REDD1)*, which are involved in DNA repair and maintaining chromosome stability. This nuclear reprogramming supports MRTX-resistant relapse in vitro. Additionally, increased NFκB2 enhances mitochondrial biogenesis and ATP production, further supporting MRTX-resistant relapse *in vitro* **D3)** To overcome MRTX resistance, we developed a novel triplet therapy strategy targeting KRAS signaling, blocking mitochondrial ATP energy conversion, and inhibiting NFκB2 simultaneously. This approach effectively treats refractory PDAC in vitro Where applicable, data are presented as means ± SEM. Statistical significance: *p < 0.05, *N* = 3. Statistical analysis: One-way ANOVA

First, we investigated a combination therapy of MRTX1133 (MRTX) [6] and Bedaquiline (BED) [7] to treat in vitro AsPC-1 human PDAC cells (48 h of treatment, Fig. 1A). Detailed information about the materials and methods is available in the Supplementary Documents. The MRTX/BED combination exhibited enhanced cytotoxic effects (a significantly higher number of trypan blue-positive dead cells and decreased cell viability validated by CCK-8 experiments) compared to MRTX or BED alone and non-treatment (Fig. 1A and Supple. Figure 1A-F). Notably, next generation RNA sequencing (transcriptomic analysis) confirmed the therapeutic effect of the MRTX/BED combination, showing that it effectively inhibits all 11 gene members of the DNA helicase CMG Complex (Fig. 1B), some of which were further validated by both qPCR (gene expression) and flow cytometry (protein expression) (Supple. Figure 2). These helicases are essential for licensing DNA replication and initiating cell cycle progression [8]. This inhibition resulted in significantly reduced Ki67-positive proliferation of AsPC-1 cells (Fig. 1C).

To uncover the refractory mechanisms associated with MRTX/BED, we conducted multiomic experimental approaches. Consistent with recent findings on *KRAS*^*G12D*^-driven cellular reprograming [9], transcriptomic analysis revealed no significant changes in *KRAS* gene expression. However, the combination therapy induced significant pro-survival nuclear reprogramming, characterized by the upregulation of prognostic genes (Supple. Figure 3 A-3B). Similar to our recent findings on NFκB2-driven drug-resistance mechanisms in acute myeloid leukemia (AML) [4], transcriptomic analysis revealed significantly increased expressions of *NFκB2* (47.22 TPM in non-treatment controls versus 130.21 TPM in combination therapy) (qPCR confirmation, Supple. Figure 3 C) along with its transcriptional coactivator *RELB* and inducer *NIK (MAP3K14)* (3.83 TPM in non-treatment controls versus 6.39 TPM in combination therapy, Fig. 1D). Additionally, gain- and loss-of-function tests of newly generated AsPC-1 transgenic cell lines demonstrated that NFκB2 promotes the genetic and protein activation of the DNA damage-induced transcript (DDIT) family members (Fig. 1E-I), which support tumor chromosomal integrity and DNA repair mechanisms [10]. NFκB2 was also found localized in the mitochondria of AsPC-1 cells (Supple. Figure 3D), consistent with our previous report of its localization in the mitochondria of an AML cell line [4], suggesting a role in malignancies (solid and blood).

To access the clinical relevance of the identified drug-resistant genes and their prognostic value, we analyzed transcriptomic datasets from approximately 13,704 PDAC patients with next-generation sequencing (NGS) data from Caris Life Sciences (Fig. 2A), and NCI-TCGA database (Supple. Figure 4). Real-world data analysis of overall survival (OS) revealed that low transcriptomic expression of all 11 DNA helicase CMG complex members was significantly associated with prolonged survival (e.g., low *CDC45* expression and low *GINS2* expression with greater than 8 months longer overall survival) in PDAC patients with *KRAS*^*G12*^ mutations (*N* = 9,717; *P* < 0.00001) (Plots and Table, Fig. 2A). These real-world findings highlight the potential of targeting the undruggable *KRAS* signaling pathway (Table, Fig. 2A) and underscore the importance of focusing on the DNA helicase CMG complex to extend OS in PDAC patients.

Building on the NFκB2-driven drug-resistance mechanisms observed in PDAC, AML [4] and metastatic breast and lung cancers [11], we developed a triplet therapy to address both metabolic and genomic plasticity. This approach comprises: (1) targeting intracellular KRAS signaling pathways with MRTX1133, (2) blocking mitochondrial ATP synthase and oxidative phosphorylation (OXPHOS) using Bedaquiline, and (3) disrupting NFκB2-mediated mitochondrial-nuclear reprogramming with SN52 (Fig. 2D1-D3). This novel triplet therapy exhibited robust cytotoxic effects against AsPC-1 cells in vitro (Supple. Figure 5) by effectively suppressing mitochondrial OXPHOS, a key ATP bioenergetic production process in PDAC tumor cells (Fig. 2B-C and Supple. Figure 6). Furthermore, we replicated our AsPC-1 results in another PDAC cell line, PANC-1, through flow cytometry, qPCR, and Seahorse experiments (Supple. Figure 7).

## Discussion

As the most frequently mutated oncogene in human cancers, *KRAS* has garnered substantial attention, particularly with recent advances in the development of *KRAS*^*G12C*^ and *KRAS*^*G12D*^ inhibitors. However, clinical trials of these inhibitors have revealed robust drug resistance mechanisms [9, 12].

While controlling abnormal cell division remains a primary goal of cancer treatment, pharmacological progress in inhibiting DNA helicases (essential for licensing DNA replication) has lagged behind [13]. In this study, we demonstrate for the first time in vitro that MRTX/BED disrupts the DNA helicase CMG complex and controls PDAC cell proliferation, suggesting that our strategy of combining a *KRAS* inhibitor with tumor metabolism and ATP-production targeting approaches could potentially improve overall survival in PDAC patients with *KRAS*^*G12*^ mutations (Fig. 2A). Consistent with the foregoing, a new class of inhibitors has been designed to competitively target ATP-binding sites in human CMG helicases and effectively suppressed tumor cell growth and DNA replication in vitro [14].

Currently, targeting the DNA damage repair (DDR) pathway (inhibiting genomic reprogramming) [15] has been proposed as a more effective strategy than vertical signaling-targeted approaches, which focus on inhibiting the cytoplasmic effector networks of the RAS family [16]. This shift aims to better suppress the “undruggable” *KRAS* signaling axis in aggressive PDAC. Here, we also successfully targeted an NFκB2-driven genomic reprogramming in MRTX/BED-resistant PDAC cells (Fig. 2D), a finding which addresses concerns raised in prior studies that identified elevated NFκB2-mediated DDR pathways in blood and solid cancers [10, 11].

However, this study has several limitations due to time & resource constraints. Future studies should rigorously evaluate the pathway-specific interactions of the three drugs in vitro, as well as assess the treatment efficacy, optimal dosage, and off-target toxicity of mono-, double-, and triple-combination MRTX/BED/SN52 treatments in primary patient tumor-derived PDAC murine models.

## Conclusions

Pancreatic cancer remains one of the most lethal malignancies. Here, we identified novel prognostic biomarkers, including members of the DNA helicase CMG complex (e.g., CDC45 and GINS2) and NFκB2-mediated genes such as *TRIB3* and *S100P*, as well as potential therapeutic targets (Fig. 2A and Supple. Figure 4). We demonstrated that NFκB2-driven transcriptional programs play a pivotal role in establishing drug-resistant cell states across in vitro models of pancreatic cancer (Fig. 2D1-3) and leukemia. Targeting the DNA helicase CMG complex and the NFκB2-driven transcriptional axis represents a promising therapeutic strategy to overcome drug resistance in refractory solid and hematologic cancers, offering new hope for improving clinical outcomes.

## Electronic supplementary material

Below is the link to the electronic supplementary material.

Supplementary Material

## Data Availability

All original datasets are presented in this article and supplementary materials..

## Abbreviations

PDAC: Pancreatic ductal adenocarcinoma
NFκB2: Nuclear Factor-kappa B subunit 2
DDIT: DNA damage-induced transcript
KRAS: Kirsten rat sarcoma viral oncogene
KRAS-I: KRAS inhibitor
MRTX: MRTX1133
BED: Bedaquiline
CMG complex: CDC45-MCM-GINS DNA Helicase
ATF3: Activating transcription factor 3
ATF4: Activating transcription factor 4
BCL2: BCL-2 apoptosis regulator
BCL3: B-cell lymphoma 3-encoded protein
MAP3K14 (also known as NIK: NFκB–inducing kinase): Mitogen-activated protein kinase, kinase, kinase 14
FC: Flow Cytometry
AML: Acute Myeloid Leukemia
PSMA4: Proteasome subunit alpha type-4
S100P: S100 calcium-binding protein P
TCGA: The Cancer Genome Atlas
TRIB3: Tribbles pseudokinase 3
