# Supplementary material for: Targeting DNA helicase CMG complex and NFκB2-driven drug-resistant transcriptional axis to effectively treat KRASG12D-mutated pancreatic cancer
Source: Exp Hematol Oncol. 2025 May 26;14:79. doi: 10.1186/s40164-025-00669-w (PMC12105384; doi:10.1186/s40164-025-00669-w)
Supplement: Supplementary file 1 — Supplementary Material [file 40164_2025_669_MOESM2_ESM.docx]

**Targeting DNA Helicase CMG Complex and NFκB2-Driven Drug-Resistant Transcriptional Axis to Effectively Treat KRAS^G12D^-Mutated Pancreatic Cancer**

Jeffrey Xiao^1#^, Joshua Kim^1#^, Brandon Park^1#^, David J. Baylink^1^, Cedric Kwon^1^, Victoria Tran^1^, Scott Lee^2^, Kevin Codorniz^2^, Laren Tan^3^, Pamela Lobo Moreno^3^, Amy Schill-Depew^3^, Saied Mirshahidi^4,5,6^, David De Semir^7^, Diana Hanna^8^, Kiran Naqvi^9^, Huynh Cao^5, 6^, Chien-Shing Chen^5, 6^, Joanne Xiu^7^, Heinz-Josef Lenz^8^, Hamid Mirshahidi^5, 6^, Mark E Reeves^5, 6^, Yi Xu^1, 5, 6 @^

**^@^Corresponding author:** Yi (David) Xu, MD, PhD**,** Loma Linda University Medical Center & Loma Linda University Cancer Center**.** Phone: 909-65-15887. Email: [dyxu@llu.edu](mailto:dyxu@llu.edu)

**Materials and Methods**

The list of reagents and transgenic cell lines including manufacturers and catalogs of antibodies, kits, primers, and lentiviral plasmids are available in the **Supplementary Tables 1-2**. Replicates (N=3) were performed for all experiments.

**Cell Culture**

AsPC-1 (ATCC CRL-1682) and PANC-1 (ATCC CRL-1469) are human-derived pancreatic cancer cell lines with the KRAS^G12D^-mutation. The AsPC-1 cell line was cultured in RPMI-1640 medium (Hyclone, Thermo Scientiﬁc), supplemented with 10% heat-inactivated fetal bovine serum (FBS, HyClone) and 1% penicillin/streptomycin. Cells were grown at 37°C in a humidiﬁed atmosphere containing 5% CO_2_. The PANC-1 cell line was cultured in Dulbecco’s Modified Eagle’s Medium (Gibco DMEM), supplemented with 10% heat-inactivated fetal bovine serum (FBS, HyClone)

***In Vitro* Treatment of AsPC-1 and PANC-1 cells**

The list of inhibitors (I), abbreviations, manufacturers, and catalog # is found in the **Supplementary Table 1**. MRTX1133 (MRTX) is an inhibitor of KRAS^G12D^ mutation. As a single agent to treat blasts *in vitro*, a single dose of 100 nM MRTX was added to 1x10^5^ AsPC-1 or PANC-1 cells in 48-well plates based on the previous report^1^. As combination agents to treat blasts *in vitro*, one dose of 100 nM MRTX with one dose of either 20 µM Bedaquiline (BED)^2^, and/or 15 µM SN52 (NFκB2-I)^3^ were added to 1 x 10^5^ AsPC-1 or PANC-1 cells for each experimental group in 48-well plates. Two days after the one dose treatment, cells were collected for analyses.

**NFκB2-overexpressed AsPC-1 cell lines**

The transgenic NFκB2-overexpressed AsPC-1 cell line was newly generated in this study by using the previously reported method^3^. Briefly, the lentiviral transfer plasmids contain a full-length open reading frame (ORF) of human NFκB2 (NM_001077494.3, GeneCopoeia catalog#: EX-Z4293-Lv225). A GFP empty vector (GeneCopoeia catalog#: EX-NEG-Lv225) was used as the vector control. The positively transduced AsPC-1 cells were purified by puromycin (2 μg/ml) and experimentally validated via microscopic and qPCR analyses of gene expression.

**NFκB2-knockdown AsPC-1 cell lines**

A short hairpin RNA (shRNA) was used to knockdown NFκB2 (p100/52) in AsPC-1 cells. AsPC-1 cells were grown in 24-well plates and transduced with human shRNA-NFκB2 lentiviral particles, which contain 4 sets of unique 29mer target-specific shRNA targeting NFκB2 (P100/P52) (Catalog#: TL311187V, OriGene, Rockville, MD, USA). AsPC-1 cells transduced with scrambled shRNA lentiviral particles were used as control (shRNA-Control). Positively transduced AsPC-1 cells were selected by puromycin (2 μg/mL). The qPCR experiments were performed to confirm NFκB2 knockdown and examine genetic changes.

**NFκB2 (P100)-eGFP reporter AsPC-1 cell lines**

The lentiviral transfer plasmid (P100-eGFP reporter) containing NFκB2 (NM_001077494.3) fused with eGFP were reported previously^3^, which is controlled by the EF1a promoter (custom-built by GeneCopoeia with catalog#: CS-Z4293-Lv224-01 or CS-Z4293-Lv224-03: without mCherry). AsPC-1 cells were lentivirally transduced to generate P100-eGFP (fusion) reporter AsPC-1 cell lines, which were further validated by microscopic and qPCR analyses.

**Flow Cytometry (FC)**

Cells were harvested and examined for the expression of viability dye, cell surface biomarkers and intracellular proteins by multichromatic FC as previously described^4^. The viability dye used in this study is Fixable Viability Dye eFluor™ 780 (eBioscience Cat#: 65-0865-14). Briefly, for FC staining, after the staining of viability dye, about 1 x 10^4^ ~ 10^6^ cells in 100 µl FC buffer (PBS containing 1% FBS and 0.05% sodium azide) were stained with various fluorescence-conjugated antibodies specific for the desired cell surface or intracellular proteins at 4^o^C. Concentrations of antibodies and dyes were applied according to the manufacturers’ recommendations. Finally, the stained cells were detected on the BD FACSAria II. FC Data was analyzed using the FlowJo software (Tree Star Inc., Ashland, OR).

**RNA-sequencing and transcriptomic analysis**

AsPC-1 cells were treated for 48 hours with one dose of combination of 100 nM MRTX1133 and 20 µM Bedaquiline as the treatment group (N=3) and without treatment as the control (N=3). High quality total RNA was isolated from collected cells by RNeasy Mini Kit (Qiagen). RNA samples were sent to BGI Americas for bulk RNA sequencing (RNA-seq) and data processing/bioinformatics analysis. The y-axis of the gene expression (TPM, transcripts per million) diagrams represents the absolute abundance of transcripts and serves as a normalization method for quantifying gene expression.

**RNA Isolation and** **Real-Time Polymerase Chain Reaction (qPCR) Analysis**

RNA isolation and qPCR analysis of gene expressions were performed as previously described^5^. Total RNAs were isolated using the RNeasy Micro Kit (Qiagen) according to the manufacturer’s instructions. First-strand cDNA was synthesized using the SuperScript III Reverse Transcriptase (Invitrogen; Life Technologies). With an Applied Biosystems 7900HT Real-Time PCR machine, qPCR was performed and analyzed. Primers used in this study are available in **Supplementary Table 2**. The PCR conditions were 10 minutes at 95°C followed by 40 cycles of 10 seconds at 95°C and 30 seconds at 60°C. The relative expression level of a gene was determined using the ΔΔCt method and normalized to β-actin.

**Imaging Acquisition**

Trypan blue staining (Gibco^TM^ 15250061) was performed according to the manufacturers’ recommendations. Imaging acquisitions were performed as previously described^6^. Phase-bright images were taken using an Olympus 1X71 inverted microscope and were processed using an Olympus cellSens Dimension 1.15 Imaging Software.

**Cell Counting Kit-8 (CCK-8) Assay**

CCK-8 Kit (Catalog No. K1018, APExBIO, Houston, Texas) was used to evaluate the cytotoxic effect of the different treatment groups in a 96 well plate. The procedure was performed according to the manufacturers’ recommendations. Briefly, 10 μL of CCK-8 reagent was added to each well for incubating 4 hours. The absorbance was measured at 450 nm with a microplate reader.

**Seahorse Assay**

Mitochondrial oxygen consumption rate (OCR) in AsPC-1 and PANC-1 cells of different experimental groups (48-hour treatment) including 1) NO TX, 2) 100 nM MRTX1133 (MRTX), 3) 20µM Bedaquiline (BED), 4) 15 µM SN52, 5) 100 nM MRTX + 20 µM BED, and 6) 100 nM MRTX + 20 µM BED + 15 µM SN52 were analyzed using Seahorse XFe24 Analyzer (Agilent, Santa Clara, CA). Following basal OCR readings, OCR was measured with oligomycin (6.25 µmol/L), FCCP (5 µmol/L) and rotenone/antimycin A (1.25 µmol/L) using Cell Mito Stress Test Kit (Agilent, Santa Clara, CA). The experimental procedure and drug concentrations were applied by following the reported protocols^7^. Six OCR parameters were calculated from the bioenergetic profile: basal OCR, ATP-linked OCR, proton leak OCR, maximal OCR, spare respiratory capacity and non-mitochondrial OCR according to the manufacturer's protocol. For AsPC-1 cells, total protein levels in each well were used to normalize readings from Seahorse XFe24 Analyzer. For PANC-1 cells, total number of cells in each well were used to normalize readings from Seahorse XFe24 Analyzer.

**Data collection and processing (TCGA)**

The Cancer Genome Atlas Program (TCGA) database (https://portal.gdc.cancer.gov/) was utilized to obtain transcriptomic data of primary PDAC tumors (without treatments). This dataset includes matched clinical and survival data for PDAC patients from the TCGA cohort, encompassing variables such as gender, age, pathological stage, and prognosis information. Patient cohort selection was based on TCGA gene expression levels (e.g., low expression or high expression of DDIT4) that matched or closely aligned with our own transcriptomic data of non-treatment or MRTX/BED treatment groups, respectively. Bioinformatic analyses and graphical outputs, such as Kaplan Meier (KM) survival curves for TCGA datasets, were generated using SRplot^8^.

**Kaplan-Meier curves data (Caris Life Sciences)**

Real-world clinical data were obtained from insurance claims. Real-world overall survival (OS) was defined as the period from tissue collection to the date of the patient’s last known clinical activity. Kaplan-Meier survival estimates were generated for cohorts defined by molecular characteristics and treatments. Hazard ratios (HR) were computed utilizing the Cox proportional hazards model, and significant differences in survival times were assessed with the log-rank test, where *P* < 0.05 was considered significant.

**Statistical analysis**

Statistical analyses were performed with GraphPad Prism software (San Diego, CA, USA). The quantitative analyses were analyzed using a one-tailed or two-tailed, unpaired *t*-test for comparison of two groups, or one-way or two-way ANOVA test for comparison of three or more groups. All values were presented as mean ± SEM. Results were considered statistically significant when the p-value was <0.05.

**Supplementary** **Table 1: List of Reagents used in this study.**

| **List of Reagents** | | | | |
| --- | --- | --- | --- | --- |
| **Antibody/Reagents** | **Abbreviation/Name in the text** | **Cat. #** | **Company** | **Species Reactivity** |
| **Viability Dye eFluor™ 780** | Viability Dye | 65-0865-14 | eBioscience |  |
| **CD44-PE/Cyanine7** | CD44 | 338816 | Biolegend | Human |
| **Ki67-APC** | Ki67 | 350513 | Biolegend | Human |
| **Ki67-PE** | Ki67 | 350504 | Biolegend | Human |
| **REDD-1-PE** | DDIT4 | SC-271158 | Santa Cruz Biotechnology | Human |
| **CDC45-PE** | CDC45 | SC-55569 | Santa Cruz Biotechnology | Human |
| **MCM3-FITC** | MCM3 | SC-390480 | Santa Cruz Biotechnology | Human |
| **FITC IgG Isotype Control** | FITC | 400107 | Biolegend | Mouse |
| **PE IgG Isotype Control** | PE | 400111 | Biolegend | Mouse |
| **APC IgG Isotype Control** | APC | 400121 | Biolegend | Mouse |
| **MRTX1133** | MRTX | HY-134813 | MedChemExpress |  |
| **Bedaquiline** | BED | HY-14881 | MedChemExpress |  |
| **SN52** |  | HY-P3229 | MedChemExpress |  |
| **Cell Counting Kit-8 (CCK-8)** |  | K1018 | APExBio |  |
| **Seahorse XF Cell Mito Stress Test Kit** |  | 103015-100 | Agilent |  |

**Supplementary** **Table 2: List of Primers (OriGene) used in this study.**

| **#** | **Name (HUMAN)** | **Forward Sequence** | **Reverse Sequence** |
| --- | --- | --- | --- |
| 1 | **NFκB2** | GGCAGACCAGTGTCATTGAGCA | CAGCAGAAAGCTCACCACACTC |
| 2 | **DDIT4 (REDD1)** | GTTTGACCGCTCCACGAGCCT | GCACACAAGTGTTCATCCTCAGG |
| 3 | **CDC45** | TGGATGCTGTCCAAGGACCTGA | CAGGACACCAACATCAGTCACG |
| 4 | **MCM5** | GACTTACTCGCCGAGGAGACAT | TGCTGCCTTTCCCAGACGTGTA |
| 5 | **GINS1**  **(PSF1)** | GCAAAGTCAGGTGGACGAAGTG | CTGATCCGAAGCAAGCGGTCAT |
| 6 | **β-Actin** | CACCATTGGCAATGAGCGGTTC | AGGTCTTTGCGGATGTCCACGT |

**Supplementary Figures and** **Figure Legends:**


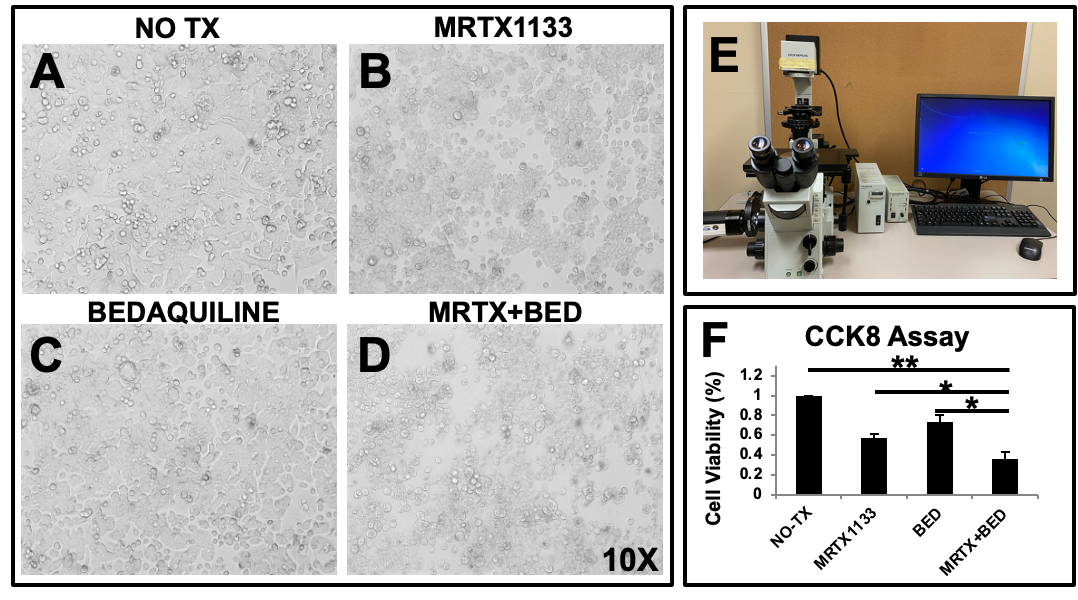


**Supplementary Figure 1. The combination of MRTX1133 (MRTX) and Bedaquiline (BED) effectively treats AsPC-1 cells *in vitro.***

***A-D)*** Representative phase-bright images of different experimental groups with NO TX, 100 nM MRTX1133, 20 µM Bedaquiline (BED), and 100 nM MRTX + 20 µM BED on AsPC-1 cells (48-hour treatment). Compared to non-treated AsPC-1 cells, which firmly adhered to the bottom of the dish and displayed heterogeneous morphologies, we observed numerous dying AsPC-1 cells with disrupted morphology, debris, and floating cell populations in all treatment groups including the MRTX/BED combination or single MRTX or BED regimens.

***E)*** Image of the Olympus 1X71 inverted microscope used to take the phase-bright and fluorescent images.

***F)*** Cell viability (%) was detected by CCK-8 assay between NO-TX control group, MRTX, BED, and MRTX + BED experimental groups.

Where applicable, data are presented as means ± SEM. *P<0.05, **P<0.01, N=3.

Statistical analysis: One-way ANOVA.


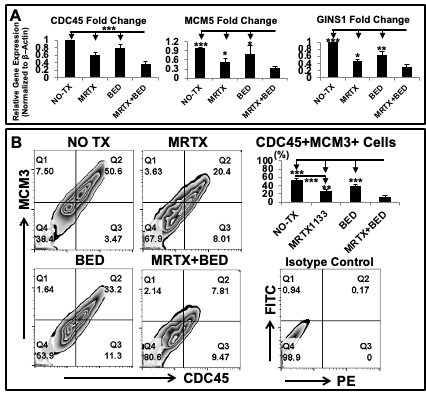


**Supplementary Figure 2. MRTX/BED treatment effectively suppresses AsPC-1 DNA Helicase gene and protein expression.**

***A)*** The gene expression levels of *CDC45*, *MCM5*, and *GINS1* were analyzed by qPCR. The mRNA expression data show the fold change (normalized to β-actin) of *CDC45*, *MCM5*, and *GINS1* across different treatment groups, including 100 nM MRTX-treated, 20 µM BED-treated, and 100 nM MRTX + 20 µM BED-treated cells, versus non-treatment (NO-TX) AsPC-1 cells (48-hour treatment);

***B)*** Representative flow cytometry (FC) contour plots of AsPC-1 cells from different experimental groups (48-hour treatment): NO TX (no treatment), 100 nM MRTX1133 (MRTX), 20 µM Bedaquiline (BED), and 100 nM MRTX + 20 µM BED, respectively; **Right upper panel:** Cumulative data showing a significant decrease in the percentage of CDC45+MCM3+ cells in MRTX/BED (TX) groups compared to non-treatment (Control) groups, 100 nM MRTX-treated, and 20 µM BED-treated groups; **Right lower panel:** Representative FC plot of the isotype control stained with FITC and PE.

Where applicable, data are presented as means ± SEM. *P<0.05, **P<0.01, ***P<0.005, N=3.

Statistical analysis: One-way ANOVA.

**
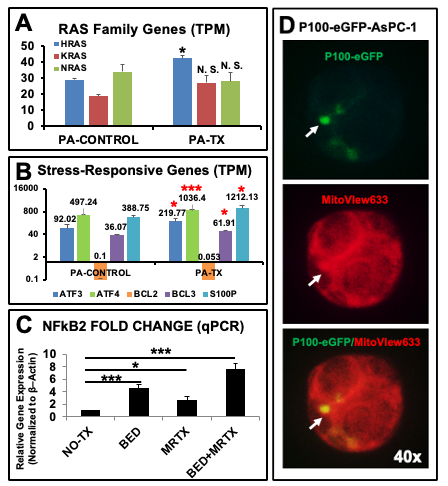
**

**Supplementary Figure 3. The MRTX/BED combination therapy promotes the expressions of stress-responsive genes and pro-survival NFκB2.**

***A)*** Cumulative RNA-seq data (TPM) of *RAS* family genes between MRTX/BED combination (TX) groups and non-treatment (Control) groups;

Consistent with recent findings on resistant mechanisms to both sotorasib (the first FDA-approved KRAS^G12C^ inhibitor) and MRTX^9,10^, our transcriptomic analysis revealed no significant changes in the gene expression of *KRAS* (although with a trend of increased expression) or *NRAS*, along with minor increase in HRAS in MRTX/BED-treated AsPC-1 cells (**Fig. 3A**). It’s possible that the pervasive *KRAS* signaling axis enables surviving AsPC-1 cells to resist treatment through KRAS^G12D^-driven cellular reprograming^11^. However, different from the resistant mechanism observed in MRTX monotherapy where activated Pan-ERBB (receptor tyrosine kinase) signaling pathways play essential roles in KRAS^G12D^-inhibitor-resistance in PDAC^12^, our RNA-seq results revealed a significant reduction (P<0.05) in the gene expression of ERBB2 (HER-2) and ERBB3 (HER-3) as well as a minor reduction (P=~0.08) of ERBB1 (EGFR, HER-1), indicating distinct mechanisms underlying resistance to the MRTX/BED combination therapy.

***B)*** Cumulative RNA-seq data (TPM) of stress-responsive gene expressions between MRTX/BED combination (TX) groups and non-treatment (Control) groups;

Previous studies on constitutive KRAS and NFκB family in PDAC demonstrated that KRAS^G12D^-activated Activator Protein 1 (AP-1) transcription factors promote a feedforward loop of IL-1a/p62 to activate and sustain the canonic RELA/NFκB1 pathway in PDAC tumor development^13^. The AP-1 machinery is a heterodimer composed of ATF, Jun and FOS protein families, which play crucial roles in cell proliferation and survival^14^. Our transcriptomic analyses revealed significantly increased expressions of activating transcription factor 3 (*ATF3*) (92.02 TPM in non-treatment controls versus 219.77 TPM in combination therapy) and *ATF4* (497.24 TPM in non-treatment controls versus 1036.4 TPM in combination therapy). ATF4 and its downstream ATF3 are key transcriptional regulators of stress-responsive genes and have been shown to promote drug resistance to tyrosine kinase inhibitors (TKIs) in hepatocellular carcinoma^15^. This suggests that surviving AsPC-1 cells actively respond to ATP depletion and treatment-induced damage by upregulating ATF3 and ATF4, which have previously been reported to reprogram cellular metabolism to promote AML relapse^16^.

The BCL-2 apoptosis regulator (BCL2) is well known as a frequent cause of drug resistance in cancer treatments and is involved in regulating caspases and other cell viability mechanisms^17^. Next, we examined the gene expression of major apoptotic regulators. Notably, there was no significant change in *BCL2* expression (which was also low in AsPC-1; 0.1 TPM in non-treatment controls versus 0.053 TPM in combination therapy); however, we discovered a significant increase in the expression of B-cell lymphoma 3-encoded protein (BCL3) (36.07 TPM in non-treatment controls versus 61.91 TPM in combination therapy). BCL3 was identified as a transcriptional coactivator of NFκB pathways (directly interacting with NFκB1/P50 and NFκB2/P52). It has since been implicated in modulating a variety of oncogenic signaling ways, including DNA damage repair and BCL3-driven therapy resistance^18^. This novel finding of enhanced *BCL3* expression suggest that BCL3 may serve as a potential central therapeutic target to overcome KRAS-I-resistance in PDAC.

S100P has been reported as a promising diagnostic marker with high sensitivity and specificity for pancreatic cancer^19,20^. The transcriptomic analysis revealed a significant increase of *S100P* expression, from 388.75 TPM in non-treatment controls to 1212.13 TPM in combination therapy groups. This elevation suggests that S100P plays a central role in treatment resistance such as EMT and distal metastasis in PDAC, as previously reported^21^. Interestingly, the interaction between S100P and RAGE (receptor for advanced glycation end products) has been shown to activate NFκB feedback loops, promoting cancer progression^22^. This indicates that S100P may serve as both a biomarker and a therapeutic target for PDAC^23^.

***C)*** Gene expression of *NFκB2* was analyzed by qPCR. Data of mRNA expressions show the fold change (normalized to *β-actin*) of NFκB2 in different treatment groups, including 20 µM BED-treated, 100 nM MRTX-treated, 100 nM MRTX + 20 µM BED, versus non-treatment (NO-TX) cells;

The qPCR analysis revealed significantly increased NFκB2 expression across all treatments compared to NO TX. Moreover, MRTX + BED-treated cells exhibited even higher NFκB2 expression (7.7-fold increase) than MRTX (2.6-fold increase) and BED (4.5-fold increase) single treatments. Altogether, consistent with our prior findings on NFκB2-driven treatment-resistant mechanisms in blood cancers^24^, both single and combination treatments activated robust compensatory responses of the noncanonical NFκB2 pathway, promoting the survival of AsPC-1 cells.

***D****)* Generation of *P100-*eGFP (fusion)-AsPC-1 cell line through the lentiviral system; P100-eGFP can be localized in mitochondria of AsCP-1 cells. The immunocytochemistry (representative fluorescent images) was performed to show the co-localization of MitoView^TM^633^+^ stained mitochondria and GFP^+^ mitochondria (white arrow) at 40x magnification;

Recently, we utilized a genetic approach of tracking P100/eGFP fusion protein and demonstrated that NFκB2 localizes to the mitochondria in MV4-11 leukemia cells and HEK-293T kidney cells^3^. To further examine whether NFκB2 is localized in the mitochondria of pancreatic tumor cells, we generated P100/eGFP fusion reporter AsPC-1 cell lines. Consistently, the P100/eGFP fusion protein was localized in the mitochondria of AsPC-1 cells, as confirmed by immunocytochemistry showing the co-localization of MitoView633^+^ mitochondria and GFP^+^ P100 (indicated by the white arrows). These findings suggest that NFκB2 may play a role in modulating mitochondrial biogenesis and ATP production as a drug-resistance mechanism in PDAC, consistent with our previous findings in AML studies^3^.

Where applicable, data are presented as means ± SEM. N.S. (not significant), *P<0.05, **P<0.01, ***P<0.005, N=3. Statistical analysis: two-tailed unpaired t test and one-way ANOVA.


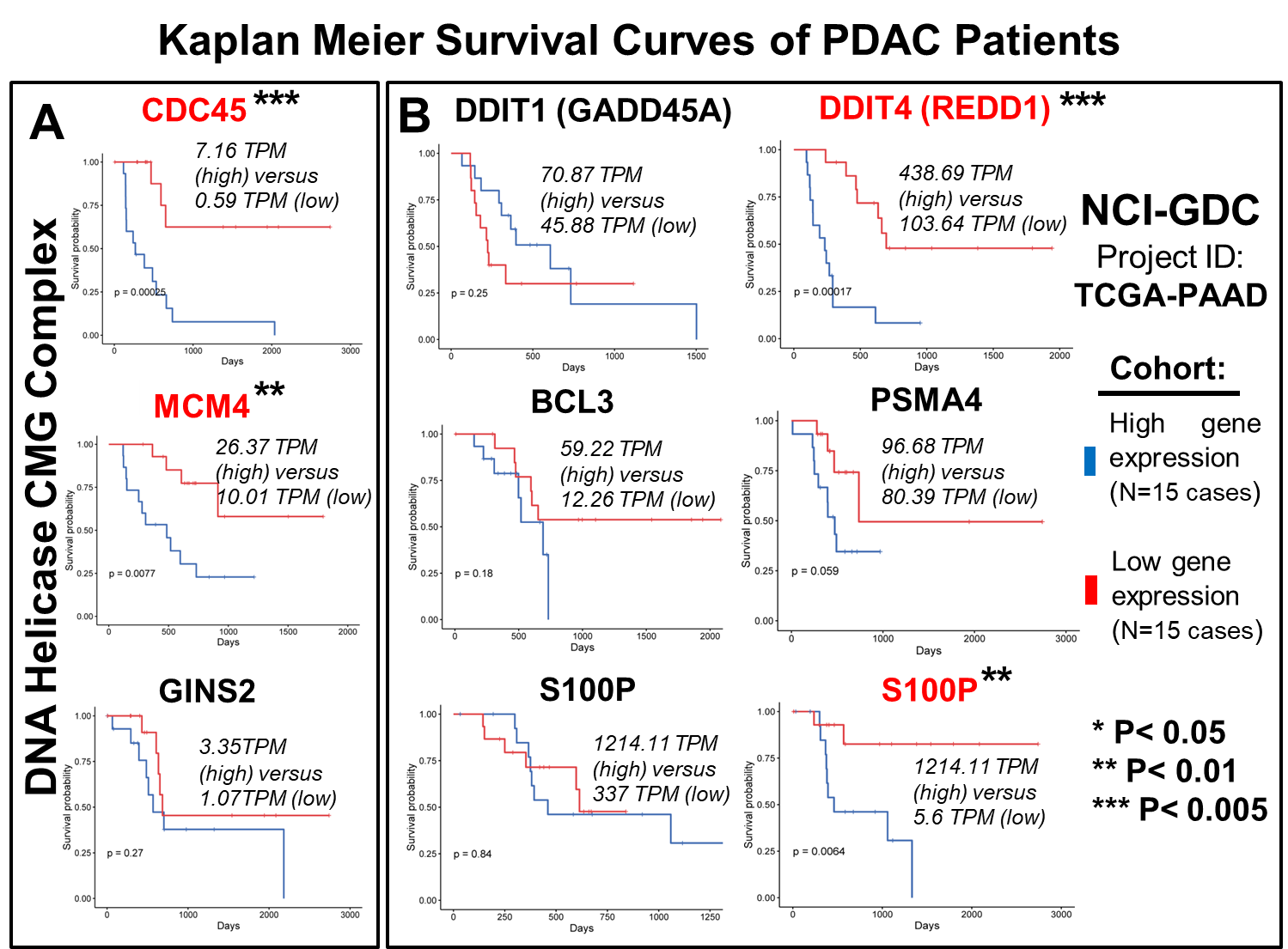


**Supplementary Figure 4. Clinical characterization of potential prognostic biomarkers for PDAC.**

***A-B)*** Kaplan-Meier survival curves of pancreatic cancer patients (NCI-GDC Project ID: TCGA-PAAD) illustrate the clinical impact of low and high expression levels of genes, including *CDC45, MCM4, GINS2, DDIT1 (GADD45A), DDIT4 (REDD1), BCL3, PSMA4* and *S100P (TPM)*. Two *S100P* plots represent different patient cohorts of low gene expression levels: Left plot: Low *S100P* expression in selected PDAC patients (N=15) closely matching our transcriptomic data (388.75 TPM); Right plot: Very low *S100P* expression in selected PDAC patients (N=15) (5.6 TPM);

To evaluate the clinical relevance of DNA helicase family gene expression in pancreatic ductal adenocarcinoma (PDAC) patients, we first analyzed data from The Cancer Genome Atlas (TCGA) database. RNA-seq data from non-treated PDAC patient samples (N = 178) were used for this analysis. Among these 178 cases, patients (N = 15 for each cohort, categorized as low-expression or high-expression) were manually selected based on their gene expression levels for the targeted gene (e.g., *CDC45*). This selection was further aligned with our own transcriptomic results comparing non-treatment (low expression of *CDC45*) versus MRTX/BED combination therapy (high expression of *CDC45*).

Bioinformatic analyses of the CMG complex genes revealed that Kaplan-Meier (KM) survival data showed a significant correlation between low expression levels of *CDC45* (0.59 TPM) and *MCM4* (10.01 TPM) with prolonged survival in PDAC patients. In contrast, high expression levels of *CDC45* (7.16 TPM) and *MCM4* (26.37 TPM) were associated with shorter survival. These findings suggest that suppressing DNA helicase family genes could improve the survival outcomes of PDAC patients, supporting the potential efficacy of MRTX/BED combination therapy *in vivo*. Additionally, *CDC45* and *MCM4* may serve as potential prognostic biomarkers for PDAC.

Notably, along the NFκB2-driven MRTX/BED-resistance axis, KM analysis also showed that high expression levels of *DDIT4 (REDD1)* (438.69 TPM) were significantly associated with shorter survival in PDAC patients compared to low expression levels (103.64 TPM). This underscores DDIT4 as a promising therapeutic target for PDAC and highlights its potential as a prognostic biomarker.

Due to the limited number of patient cases with transcript per million (TPM) values comparable to our transcriptomic data, low expression of *BCL3* and Proteasome subunit alpha type-4 (*PSMA4*, a potential biomarker for PDAC^25^) did not exhibit statistically significant benefits. However, a trend toward extended survival was observed in association with their low expression levels.

Interestingly, while modest reductions in *S100P* expression (337 TPM) did not correlate with prolonged survival, extremely low expression levels of *S100P* (5.6 TPM) were significantly associated with improved survival in PDAC patients compared to high expression levels (1214.11 TPM). Overall, these findings are consistent with prior studies showing that elevated expression of noncanonical NFκB pathways is strongly correlated with shorter metastasis-free or disease-free survival in patients with solid cancer^26^.

In summary, our real-world OS data suggests that combination therapy with MRTX1133 and Bedaquiline is a promising strategy, as it suppresses all 11 genes of the DNA helicase CMG complex, potentially improving OS for PDAC patients with KRAS^G12D^ mutation.

Where applicable, data are presented as means ± SEM. *P<0.05, **P<0.01, ***P<0.005.


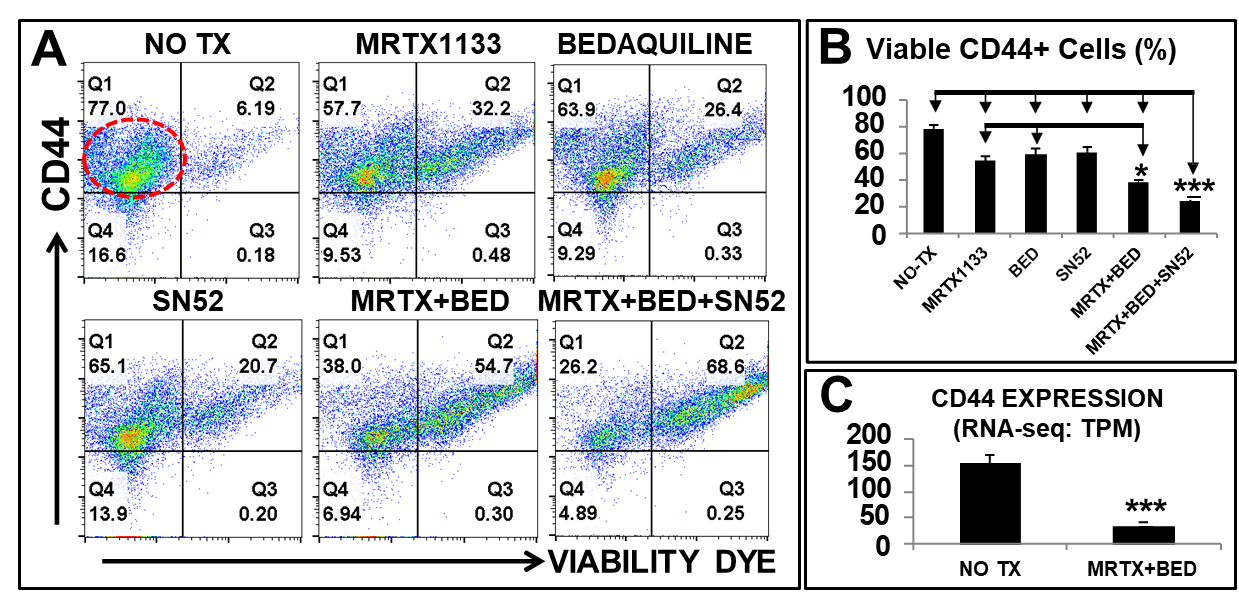


**Supplementary Figure 5. NFκB2-inhibition significantly improves the treatment efficacy of MRTX/BED combination on *in vitro* AsPC-1 cells.**

***A)*** Representative FC plots of different experimental groups (48-hour treatment) with NO TX (no treatment), 100 nM MRTX1133 (MRTX), 20 µM Bedaquiline (BED), 15 µM SN52, MRTX + BED, and MRTX + BED + SN52, respectively; Red circle indicates CD44^+^/viable (viability dye^-^) AsPC-1 cells;

***B)*** Cumulative percentage data of viable CD44+ AsPC-1 cells in different treatment groups;

***C)*** Cumulative RNA-seq data (TPM) of *CD44* gene expressions between MRTX/BED combination (TX) groups and non-treatment (Control) groups;

Based on the above evidence suggesting the central role of NFκB2 in MRTX/BED-resistant mechanisms in PDAC tumor cells, we next performed a preliminary experiment to examine whether SN52 (an NFκB2 inhibitor) can improve the treatment efficacy of the MRTX/BED combination *in vitro*. Flow cytometry analyses of the cell death biomarker (viability dye) and the PDAC tumor cell biomarker (CD44)^27^ revealed that the MRTX/BED combination therapy significantly reduced the population of viable CD44^+^AsPC-1 cells (38%) compared to the non-treatment groups (77%), single-agent MRTX (57.7%), or BED regimens (63.9%). Additionally, transcriptomic analysis confirmed that the MRTX/BED combination significantly reduced the gene expression of *CD44* (155.28 TPM in the non-treatment control versus 35.17 TPM in the combination therapy group). CD44 has been previously reported to play an essential role in gemcitabine resistance and PDAC invasion^27^. Furthermore, the addition of SN52 to the MRTX/BED combination exhibited the strongest therapeutic effect, eliminating a greater percentage of aggressive AsPC-1 cells (26.2%) compared to all other experimental groups.

Where applicable, data are presented as means ± SEM. *P<0.05, **P<0.01, ***P<0.005, N=3.

Statistical analysis: two-tailed unpaired t test and one-way ANOVA.


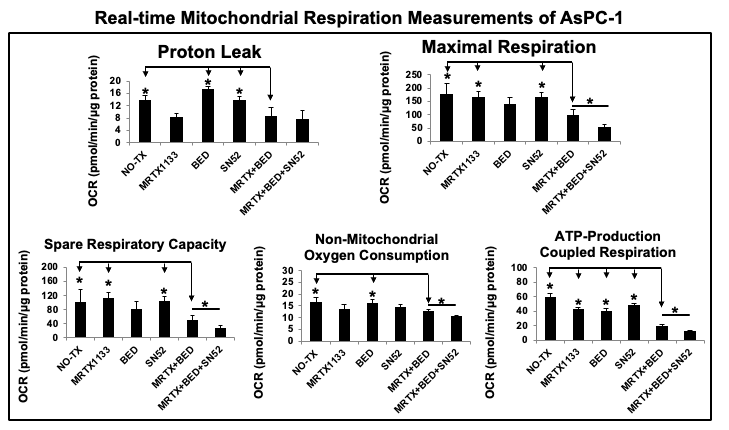


**Supplementary Figure 6. Cumulative Seahorse data of five OCR parameters across different treatment groups of AsPC-1 cells.**

Mitochondrial oxygen consumption rate (OCR) in AsPC-1 cells was analyzed using the Seahorse XFe24 Analyzer (Agilent) after 48 hours of treatment under different experimental conditions. The treatment groups included: 1) NO TX (non-treated control), 2) 100 nM MRTX1133 (MRTX), 3) 20 µM Bedaquiline (BED), 4) 15 µM SN52, 5) 100 nM MRTX + 20 µM BED, and 6) 100 nM MRTX + 20 µM BED + 15 µM SN52. Five OCR parameters were calculated from the bioenergetic profile according to the manufacturer's protocol: proton leak OCR, maximal OCR, spare respiratory capacity, non-mitochondrial OCR and ATP-linked OCR.

Where applicable, data are presented as means ± SEM. *P<0.05, N=3. Statistical analysis: One-way ANOVA.


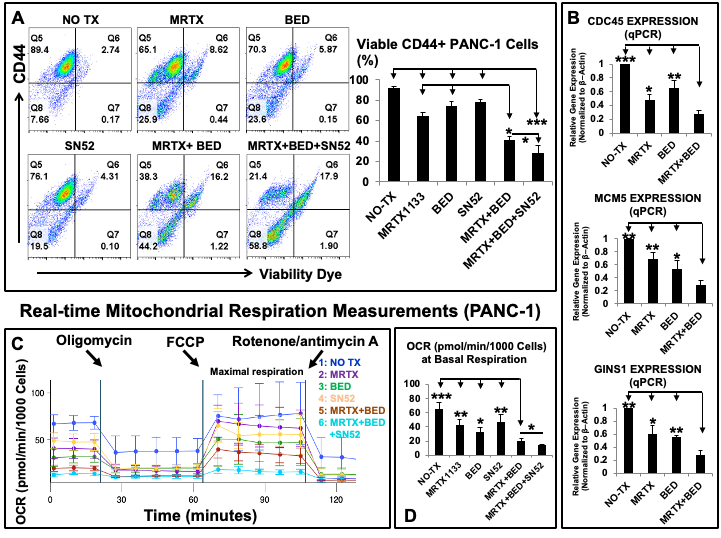


**Supplementary Figure 7. MRTX/BED/SN52 (NFκB2-Inhibitor) or MRTX/BED combination therapy effectively treats CD44+ PANC-1 cells and suppresses DNA helicase gene expression.**

***A)*** Representative FC density plots of PANC-1 cells from different experimental groups (48-hour treatment): NO TX (no treatment), 100 nM MRTX1133 (MRTX), 20 µM Bedaquiline (BED), 15 µM SN52, MRTX + BED, and MRTX + BED + SN52, respectively; **Right panel:** Cumulative data show a significant decrease in the percentage of viable CD44+ PANC-1 cells in MRTX/BED/SN52 (TX) groups compared to non-treatment (Control) groups, 100 nM MRTX-treated, 20 µM BED-treated, 15 µM SN52-treated, and MRTX + BED-treated groups;

***B)*** The gene expressions of *CDC45*, *MCM5*, and *GINS1* were analyzed by qPCR. The mRNA expression data show the fold change (normalized to β-actin) of *CDC45*, *MCM5*, and *GINS1* in different treatment groups, including 100 nM MRTX-treated, 20 µM BED-treated, and 100 nM MRTX + 20 µM BED, compared to non-treatment (NO-TX) PANC-1 cells;

***C)*** Mitochondrial oxygen consumption rate (OCR) in PANC-1 cells was analyzed using the Seahorse XFe24 Analyzer (Agilent) after 48 hours of treatment under different experimental conditions. The treatment groups included: 1) NO TX (non-treated control), 2) 100 nM MRTX1133 (MRTX), 3) 20 µM Bedaquiline (BED), 4) 15 µM SN52, 5) 100 nM MRTX + 20 µM BED, and 6) 100 nM MRTX + 20 µM BED + 15 µM SN52.

***D)*** Cumulative Seahorse data of basal OCR readings across different treatment groups. Real-time OCR traces and averaged data revealed that oxygen consumption devoted to ATP production was significantly decreased in both combination and triplet therapies at the basal respiratory stage, compared to single-agent regimens and non-treated controls.

Where applicable, data are presented as means ± SEM. *P<0.05, **P<0.01, ***P<0.005, N=3.

Statistical analysis: One-way ANOVA.


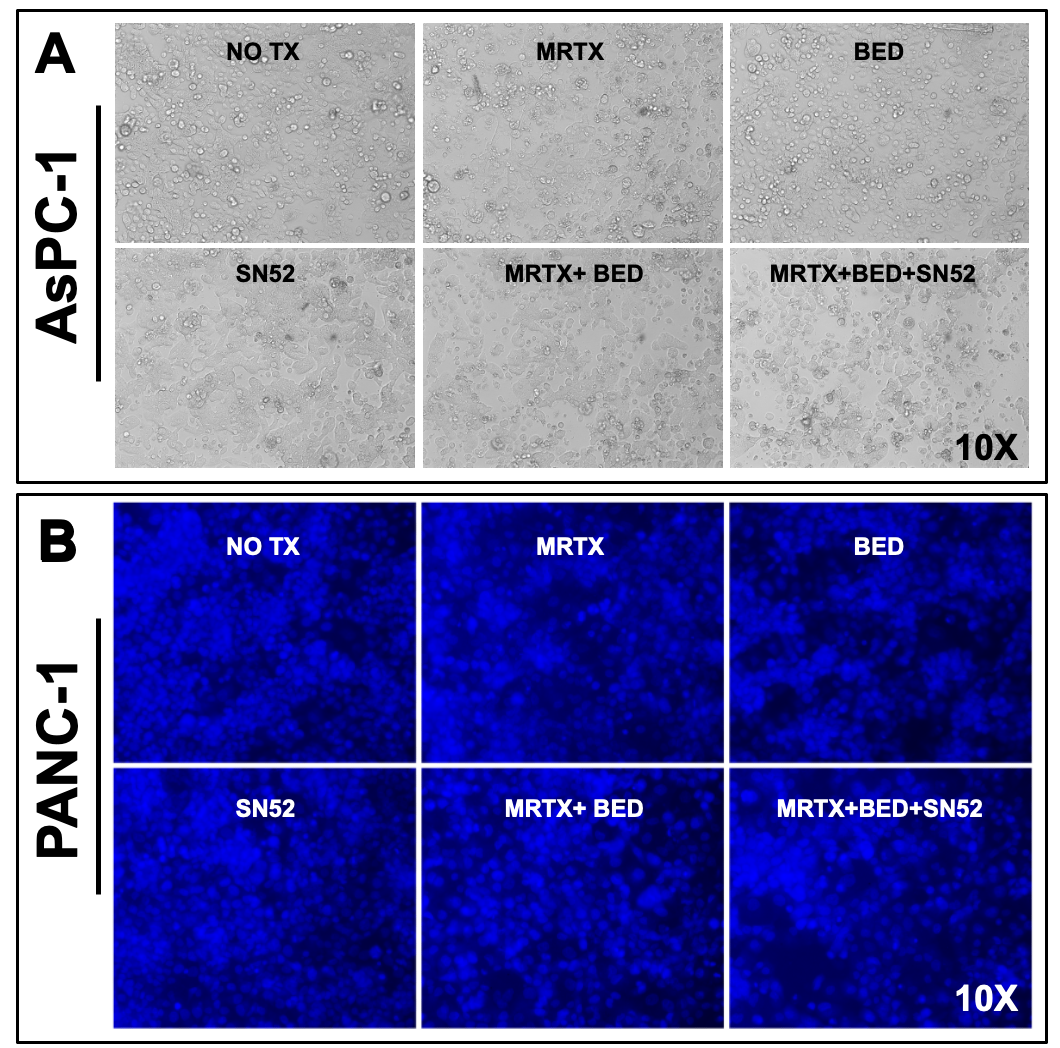


**Supplementary Figure 8. Sufficient cell density and nearly 100% confluence across all treatment groups for AsPC-1 and PANC-1 ensured reliable Seahorse assay results after combination or triplet therapies in vitro.**

***A)*** After Seahorse experiments with AsPC-1 cells, images were taken to assess cell density across different experimental groups. Phase-bright 10X images of AsPC-1 cells (after 48 hours of treatment) show the following conditions: NO TX (no treatment), 100 nM MRTX1133 (MRTX), 20 µM Bedaquiline (BED), 15 µM SN52, MRTX + BED, and MRTX + BED + SN52. The imaging confirms sufficient cell density, meeting the recommended minimum of 5,000 cells per well for all treatment groups. This indicates that the observed OCR changes in the Seahorse results were not due to insufficient cell numbers.

***B)*** After Seahorse experiments with PANC-1 cells, DAPI staining was performed to assess cell attachment, followed by imaging. Fluorescent 10X DAPI-stained images of PANC-1 cells (after 48 hours of treatment) show the following conditions: NO TX (no treatment), 100 nM MRTX1133 (MRTX), 20 µM Bedaquiline (BED), 15 µM SN52, MRTX + BED, and MRTX + BED + SN52. The imaging indicates that the majority of cells remained attached across all treatment groups, suggesting that the Seahorse experiments were conducted under nearly 100% confluence conditions.

**Reference**

1 Kumarasamy, V. *et al.* The Extracellular Niche and Tumor Microenvironment Enhance KRAS Inhibitor Efficacy in Pancreatic Cancer. *Cancer Res* **84**, 1115-1132 (2024). <https://doi.org/10.1158/0008-5472.CAN-23-2504>

2 Fiorillo, M. *et al.* Bedaquiline, an FDA-approved antibiotic, inhibits mitochondrial function and potently blocks the proliferative expansion of stem-like cancer cells (CSCs). *Aging (Albany NY)* **8**, 1593-1607 (2016). <https://doi.org/10.18632/aging.100983>

3 Xu, Y. *et al.* Discovery of NFkappaB2-Coordinated Dual Regulation of Mitochondrial and Nuclear Genomes Leads to an Effective Therapy for Acute Myeloid Leukemia. *Int J Mol Sci* **25** (2024). <https://doi.org/10.3390/ijms25158532>

4 Cao, H. *et al.* Discovery of proangiogenic CD44+mesenchymal cancer stem cells in an acute myeloid leukemia patient's bone marrow. *J Hematol Oncol* **13**, 63 (2020). <https://doi.org/10.1186/s13045-020-00899-x>

5 Xu, Y. *et al.* A novel vitamin D gene therapy for acute myeloid leukemia. *Transl Oncol* **13**, 100869 (2020). <https://doi.org/10.1016/j.tranon.2020.100869>

6 Xu, Y. *et al.* Vitamin D activates FBP1 to block the Warburg effect and modulate blast metabolism in acute myeloid leukemia. *Biomark Res* **10**, 16 (2022). <https://doi.org/10.1186/s40364-022-00367-3>

7 Song, R., Dasgupta, C., Mulder, C. & Zhang, L. MicroRNA-210 Controls Mitochondrial Metabolism and Protects Heart Function in Myocardial Infarction. *Circulation* **145**, 1140-1153 (2022). <https://doi.org/10.1161/CIRCULATIONAHA.121.056929>

8 Tang, D. *et al.* SRplot: A free online platform for data visualization and graphing. *PLoS One* **18**, e0294236 (2023). <https://doi.org/10.1371/journal.pone.0294236>

9 Awad, M. M. *et al.* Acquired Resistance to KRAS(G12C) Inhibition in Cancer. *N Engl J Med* **384**, 2382-2393 (2021). <https://doi.org/10.1056/NEJMoa2105281>

10 Dilly, J. *et al.* Mechanisms of Resistance to Oncogenic KRAS Inhibition in Pancreatic Cancer. *Cancer Discov* **14**, 2135-2161 (2024). <https://doi.org/10.1158/2159-8290.CD-24-0177>

11 Juul, N. H. *et al.* KRAS(G12D) drives lepidic adenocarcinoma through stem-cell reprogramming. *Nature* **619**, 860-867 (2023). <https://doi.org/10.1038/s41586-023-06324-w>

12 Gulay, K. C. M. *et al.* Dual Inhibition of KRASG12D and Pan-ERBB Is Synergistic in Pancreatic Ductal Adenocarcinoma. *Cancer Res* **83**, 3001-3012 (2023). <https://doi.org/10.1158/0008-5472.CAN-23-1313>

13 Ling, J. *et al.* KrasG12D-induced IKK2/beta/NF-kappaB activation by IL-1alpha and p62 feedforward loops is required for development of pancreatic ductal adenocarcinoma. *Cancer Cell* **21**, 105-120 (2012). <https://doi.org/10.1016/j.ccr.2011.12.006>

14 Karin, M., Liu, Z. & Zandi, E. AP-1 function and regulation. *Curr Opin Cell Biol* **9**, 240-246 (1997). <https://doi.org/10.1016/s0955-0674(97)80068-3>

15 Dai, Z. *et al.* Induction of IL-6Ralpha by ATF3 enhances IL-6 mediated sorafenib and regorafenib resistance in hepatocellular carcinoma. *Cancer Lett* **524**, 161-171 (2022). <https://doi.org/10.1016/j.canlet.2021.10.024>

16 Di Marcantonio, D. *et al.* ATF3 coordinates serine and nucleotide metabolism to drive cell cycle progression in acute myeloid leukemia. *Mol Cell* **81**, 2752-2764 e2756 (2021). <https://doi.org/10.1016/j.molcel.2021.05.008>

17 Garcia-Aranda, M., Perez-Ruiz, E. & Redondo, M. Bcl-2 Inhibition to Overcome Resistance to Chemo- and Immunotherapy. *Int J Mol Sci* **19** (2018). <https://doi.org/10.3390/ijms19123950>

18 Seaton, G., Smith, H., Brancale, A., Westwell, A. D. & Clarkson, R. Multifaceted roles for BCL3 in cancer: a proto-oncogene comes of age. *Mol Cancer* **23**, 7 (2024). <https://doi.org/10.1186/s12943-023-01922-8>

19 Ali, A. *et al.* Expression of KOC, S100P, mesothelin and MUC1 in pancreatico-biliary adenocarcinomas: development and utility of a potential diagnostic immunohistochemistry panel. *BMC Clin Pathol* **14**, 35 (2014). <https://doi.org/10.1186/1472-6890-14-35>

20 Hu, H. *et al.* Diagnostic value of S100P for pancreatic cancer: a meta-analysis. *Tumour Biol* **35**, 9479-9485 (2014). <https://doi.org/10.1007/s13277-014-2461-4>

21 Naidoo, K. *et al.* Proteome of formalin-fixed paraffin-embedded pancreatic ductal adenocarcinoma and lymph node metastases. *J Pathol* **226**, 756-763 (2012). <https://doi.org/10.1002/path.3959>

22 Riehl, A., Nemeth, J., Angel, P. & Hess, J. The receptor RAGE: Bridging inflammation and cancer. *Cell Commun Signal* **7**, 12 (2009). <https://doi.org/10.1186/1478-811X-7-12>

23 Arumugam, T. & Logsdon, C. D. S100P: a novel therapeutic target for cancer. *Amino Acids* **41**, 893-899 (2011). <https://doi.org/10.1007/s00726-010-0496-4>

24 Cao, H. *et al.* Targeting TKI-Activated NFKB2-MIF/CXCLs-CXCR2 Signaling Pathways in FLT3 Mutated Acute Myeloid Leukemia Reduced Blast Viability. *Biomedicines* **10** (2022). <https://doi.org/10.3390/biomedicines10051038>

25 Watcharanurak, P., Mutirangura, A., Aksornkitti, V., Bhummaphan, N. & Puttipanyalears, C. The high FKBP1A expression in WBCs as a potential screening biomarker for pancreatic cancer. *Sci Rep* **14**, 7888 (2024). <https://doi.org/10.1038/s41598-024-58324-z>

26 Bakhoum, S. F. *et al.* Chromosomal instability drives metastasis through a cytosolic DNA response. *Nature* **553**, 467-472 (2018). <https://doi.org/10.1038/nature25432>

27 Zhao, S. *et al.* CD44 Expression Level and Isoform Contributes to Pancreatic Cancer Cell Plasticity, Invasiveness, and Response to Therapy. *Clin Cancer Res* **22**, 5592-5604 (2016). <https://doi.org/10.1158/1078-0432.CCR-15-3115>
